# HIV program outcomes for Jamaica before and after “Treat All”: A population-based study using the national treatment services database

**DOI:** 10.1371/journal.pone.0255781

**Published:** 2021-08-12

**Authors:** Anya Cushnie, Ralf Reintjes, Susanna Lehtinen-Jacks, J. Peter Figueroa

**Affiliations:** 1 Unit of Health Sciences, Faculty of Social Sciences, Tampere University, Tampere, Finland; 2 Department of Health Sciences, Hamburg University of Applied Sciences, Hamburg, Germany; 3 School of Health, Care and Social Welfare, Mälardalen University, Västerås, Sweden; 4 Department of Community Health and Psychiatry, University of the West Indies, Mona, Jamaica; University of Ghana College of Health Sciences, GHANA

## Abstract

**Objective:**

The study aims to assess changes in HIV treatment outcomes for Jamaica after the implementation of the WHO Treat All strategy in January 2017, as well as identify variables associated with clinical stage at diagnosis and viral load status, in order to understand implications for enhancing the HIV clinical cascade and boosting progress towards the UNAIDS 90-90-90 targets.

**Method:**

This is a population-based study using the National Treatment Service Information System. The sample consists of persons 15 years and older, placed on treatment before and after Treat All was implemented, across all 4 regional health authorities in Jamaica. Patients were assessed for two binary outcomes: 1. stage at HIV diagnosis (early/baseline CD4 cell count ≧350 cells/mm^3^, or late/ baseline CD4 <350 cells/mm^3^), 2. viral load status achieved after ART initiation (suppressed/<1000 copies/ml or non-suppressed/ ≥1000 copies/ml). Categorical variables: age/years, gender and health regions, were investigated using multivariable logistic regression. Adjusted odds ratios and 95% confidence intervals are reported.

**Results:**

After Treat All, there was an increase in median baseline CD4 results as the proportion of late diagnoses decreased from 60% to 39%. There was a small increase in viral suppression from 76% to 80%, a decrease in baseline viral load testing from 61% to 46% and an increase in the uptake of first viral load testing after starting treatment from 13% to 19%. Males and persons 40+ years had higher odds of late diagnosis before and after Treat All.

**Conclusion:**

Jamaica’s HIV program outcomes have improved after Treat All was implemented. ART initiation time significantly decreased. Early diagnosis, viral load testing uptake and viral suppression increased. However, there is a need to implement targeted testing for men and persons over 40 years to decrease the frequency of late diagnosis.

## Introduction

As of 2018, Jamaica recorded an adult HIV prevalence of 1.5%, with an estimated 32,617 persons living with HIV (PLHIV) [1]. To improve HIV treatment outcomes, the WHO Treat All strategy was implemented in January 2017 [2]. Treat All is recognized as the primary strategy for achieving the UNAIDS 90‐90‐90 targets [3]. In 2018, Jamaica’s achievement of the UNAIDS targets was 84-53-65 [4]. Compared with the global achievement of 75-79-81 [5], Jamaica has made progress in testing/diagnosis but retention on treatment and viral suppression are lagging.

Treat All has several objectives, including early diagnosis of HIV, immediate initiation of antiretroviral therapy (ART) and viral suppression [6]. Diagnosis is the first step in HIV care and globally also the largest gap [3]. Men are disproportionately affected only receiving 30% of HIV tests [7]. Young men (20–39 years old) are also more likely than young women to be living with HIV but much less likely to take a HIV test [8]. Ensuring an effective response to ART, reducing morbidity and mortality requires increased testing coverage to decrease the chances of late diagnosis [9], defined as an initial CD4 count <350 cells/mm^3^ [10]. In 2017, 33% of Jamaicans on HIV treatment were diagnosed at a late HIV stage [2], justifying the implementation of Treat All. Sustained viral suppression is the goal of HIV treatment programs [11, 12] because of evidence showing undetectable viral load improves the health of PLHIV and makes HIV less likely to be transmitted [13]. More than half of all people with HIV globally are not virally suppressed [4]. Virologic data also provides useful information such as possible treatment failure and drug resistance [14].

The primary aim of the study is to assess characteristics of PLHIV on treatment, before and after the implementation of the new treatment strategy, by identifying factors associated with the stage at diagnosis and viral load status, in order to understand implications for enhancing the HIV treatment cascade and population based outcomes for Jamaica. Secondary objectives aim to assess changes in ART and viral load testing uptake. This paper is the first in a series of studies which will look at factors associated with improving the HIV treatment cascade and help to suggest programmatic strategies to narrow current gaps.

## Materials and methods

This secondary analysis uses the National Treatment Service Information System (TSIS2). TSIS2 is a centralized case-based database, governed by the Ministry of Health and Wellness (MoHW), Jamaica and currently being used by all HIV treatment sites in Jamaica. TSIS2 stores patient level demographic and clinical data, using over 100 variables [15]. This study assesses 8 routinely collected variables. The MoHW Internal Review Board provided ethical approval (Study No: 2017/20). The dataset was extracted by the MoHW and fully anonymized. The requirement for informed consent was waived and a confidentiality agreement signed which prohibited data sharing.

### Sample population

**Fig 1:**
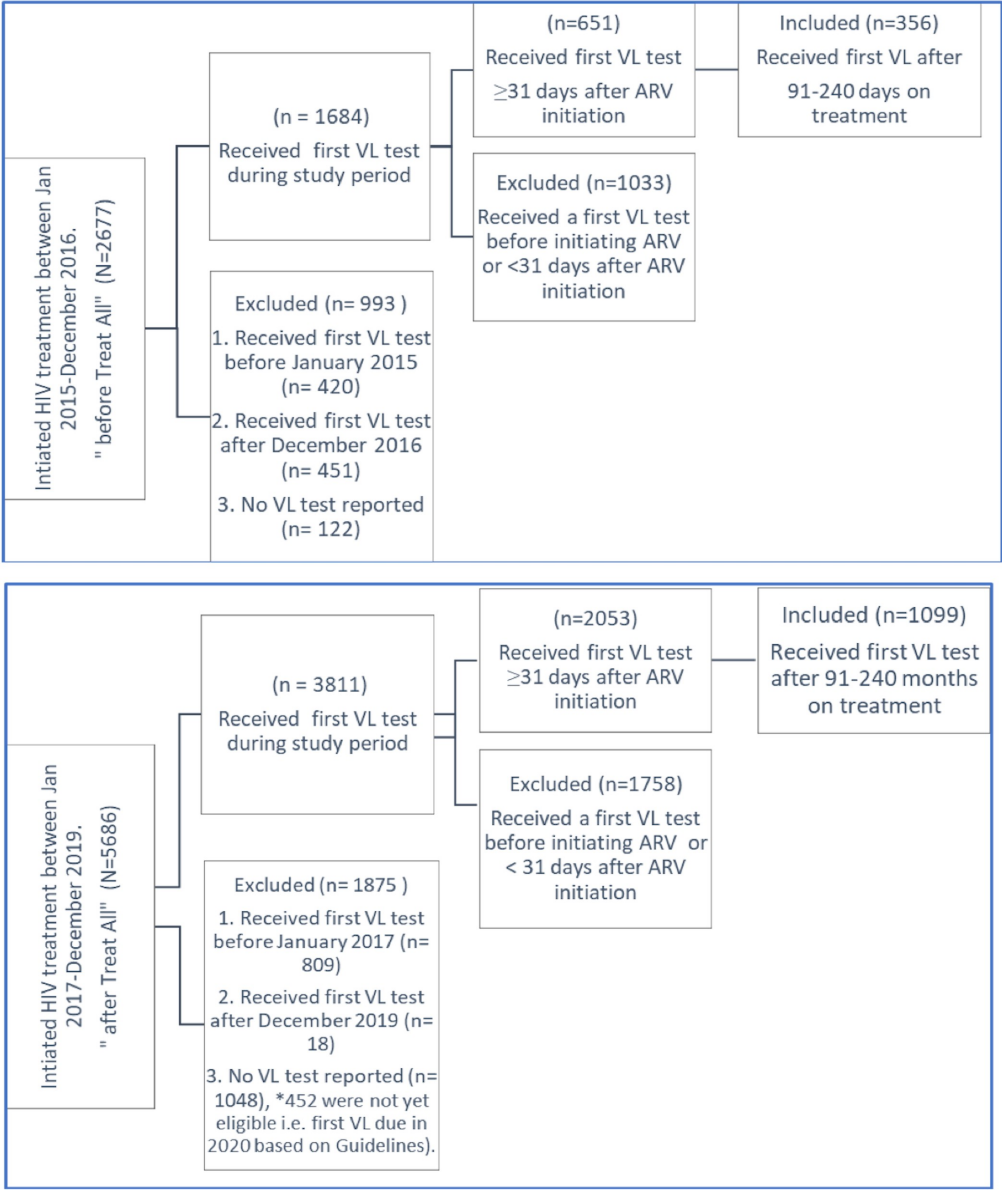
Results of inclusion criteria applied for selection of the final analytical samples.

The baseline data extracted included all PLHIV initiating HIV treatment between January 2015-December 2019. The sample was divided into two datasets representing before Treat All implementation (January 2015-December 2016) and after Treat All (January 2017-December 2019) (Table 1) and an inclusion criterion applied (Fig 1). To ensure consistency across timelines and no overlap between the two study periods, persons were excluded if they had no recorded viral load test results or if they had received a first viral load test before or after the respective study period. As Jamaica’s current national treatment guidelines stipulate, a first viral load test should be received within 6 months after ART initiation [15], inclusion for the final analytical sample was restricted to viral load measurements between 91 and 240 days after ART initiation to ensure the sample consisted of persons who may have achieved some control on treatment.

**Table 1:** Demographic and clinical characteristics for the baseline sample of persons living with HIV (PLHIV) on ART.

Variables	Levels	Before Treat All		After Treat All	
		n	%	n	%
**Gender**					
	Female	1416	52.9	3087	54.9
	Male	1259	47	2531	45.1
	*Missing*	*2*		*0*	
**Age group at ART initiation/years**					
	Mean (SD)	37.6 (13.1)		37.2 (13.7)	
	< 15 years	34	1.3	37	0.7
	15–19	130	4.8	360	6.5
	20–39	1398	52.3	2981	53.4
	40+	1109	41.5	2205	39.5
	*Missing*	*6*		*35*	
**RHA^a^**					
	SERHA	1365	51	2860	50.9
	WRHA	628	23.5	1285	22.9
	NERHA	406	15.2	729	13
	SRHA	278		744	
**HIV stage at diagnosis ^b^**					
	Median baseline CD4 result/cells/mm^3^ (IQR)	365 (373)		462 (423)	
	early diagnosis	1384	52.6	3152	64.7
	late diagnosis	1249	47.4	1718	35.3
	*Missing*	*0*		*748*	
	Median time to ART initiation/days ^c^ (IQR)	35 (552.5)		6 (348.5)	
**Viral load status after ART initiation^d^**					
	Median first viral load result/copies/mL (IQR)	7982.5 (55914.2)		743 (25975.2)	
	non-suppressed	1590	62.3	2233	48.4
	Suppressed	946	37.7	2377	51.6
	*Missing*	*123*		*1008*	
	Median time to first VL test/days^e^ (1QR)	1 (283.8)		6 (217)	

All PLHIV in Jamaica initiating HIV treatment in 2015–2016, before Treat All (N = 2677) and 2017–2019, after Treat All (N = 5618).

a. RHA-Regional Health Authority, SERHA-Southeast Regional Health Authority, SRHA–Southern Regional Health Authority, NERHA–Northeast Regional Health Authority, WRHA–Western Regional Health Authority.

b. Based on baseline CD4 test results: Patients starting ART with CD4 cell count ≧350 cells/mm3 were characterized as achieving early diagnosis, while CD4 <350 cells/mm3 was defined as late diagnosis.

c. Time/days to ART initiation is the time from first clinic date to ART initiation.

d. Based on the first viral load test after initiating treatment, followed by categorization according to the first viral load test results at that time period (<1000 copies/mL versus ≧1000 copies/mL).

e. Time/days to VL test is the time from the ART initiation date to first VL test date.

### Variables of interest

The following routinely collected variables were extracted from the TSIS2: gender (male, female), age (years), Regional Health Authority (RHA) (Southeast, Southern, Northeast and Western), first clinic date, first ART date, first CD4 cell count (cells/mm^3^), first viral load test date and result (copies/mL). Age was categorized into 3 groups (15–19, 20–39, ≥40 years) for the analyses. Two binary outcomes were assessed, each defined by the *Clinical Management of HIV Disease Guidelines* [16], as follows:

1. HIV stage at diagnosis (early or late). Patients starting ART with baseline CD4 cell count ≧350 cells/mm^3^ were characterized as achieving early diagnosis, while a baseline CD4 <350 cells/mm^3^ was defined as late diagnosis [17].
2. First viral load status achieved after starting ART (suppressed, non-suppressed). The results were categorized as suppressed <1000 copies/mL or non-suppressed ≥1000 copies/mL).

### Statistical analysis

The samples were stratified by categorical variables: age group, gender and RHA. Sample sizes were small for persons < 15 years of age, so the regression analysis focused on persons 15+ years. We analyzed time to ART initiation based on the time from the first clinic date to ART initiation date. Time to first viral load test was determined by the time from the first ART date to the first viral load test date. Jamaica’s current national treatment guidelines stipulate a first viral load test should be received within 6 months after ART initiation [16] so, inclusion for the final analytical sample was restricted to persons who received a first viral load test 91 and 240 days after ART initiation to ensure the sample consisted of persons who may have achieved some control on treatment. Mann-Whitney U test was used to assess the difference in time to ART initiation between samples. Bivariate analyses and multivariable logistic regression models were used to analyze associations between the demographic variables with the two outcomes (HIV stage at diagnosis, viral suppression), as demonstrated in Model A [18].


logit(p)=β0+β1(agegrp)+β2(RHA)+β3(gender)ModelA

Additionally, the association between HIV stage at diagnosis and viral suppression was also assessed, as shown in Model B.


logit(p)=β0∼B4(HIVstageatdiagnosis)ModelB

No mathematical correction was made for multiple comparisons. Both datasets were analyzed separately using *R*, version 3.5.3 [19] and the final fit package was used to generate regression results and plots [20]. The full reproducible code is provided in S1 File.

## Results

The baseline samples consisted of 2677 PLHIV initiating treatment before Treat All and 5686 after Treat All. Table 1 provides a descriptive analysis of the initial sample and shows that gender was evenly distributed within samples, with a small increase in the proportion of females diagnosed and accessing viral load testing after Treat All (52.9% to 54.9%). The mean ages were similar for both samples, with most persons from the 20–39 years and 40+ years age groups. Most persons accessing care lived in the area of SERHA before and after Treat All. Median baseline CD4 results increased from 365 cells/mm^3^ before Treat All to 462 cells/mm^3^ after Treat All, and the proportion of persons diagnosed early increased from 52.6% to 64.7%. There was also an increase in viral suppression from 37.7% to 51.6% and similarly a noticeable reduction in median first viral load results. A significant decrease in ART initiation time after Treat All (median 35 days vs. 6 days, p<0.001) was also reflected.

**Fig 2:**
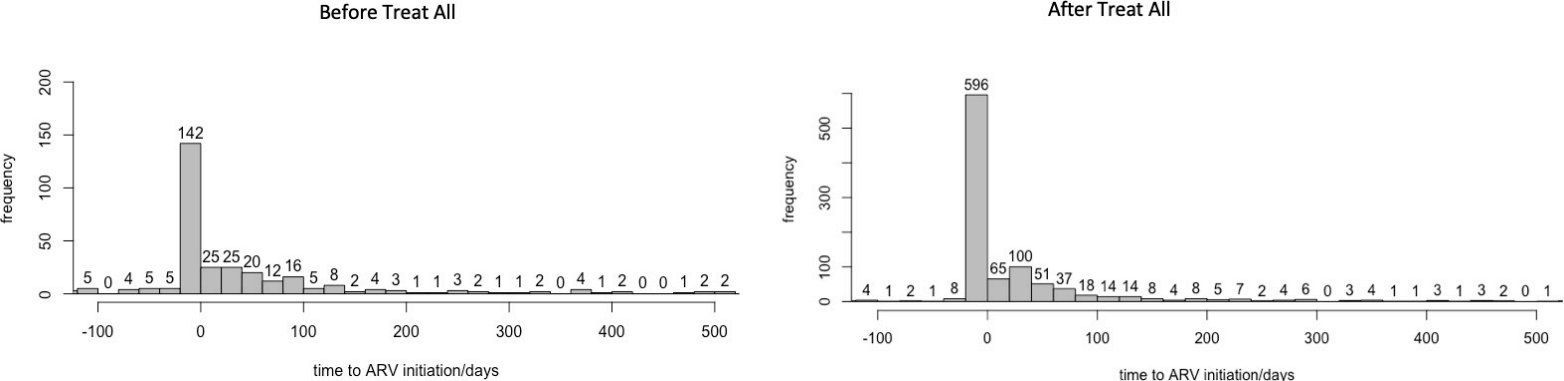
Histograms showing time to ARV initiation/days for baseline samples before Treat All in 2015–2016 and after Treat All in 2017–2019.

Fig 1 shows that before Treat All, 61% (n = 1033) received a first viral load test before ART initiation (baseline viral load test) compared to 46% (n = 1758) after Treat All. This is congruent with the national treatment guidelines and the Treat All strategy, which both recommend reduced baseline viral load testing [10, 17]. The final analytical samples consisted of PLHIV who started HIV treatment between January 2015-December 2019 and received the first viral load test 91–240 days after starting ART. This was approximately 54% of persons who had received a first viral load test in both scenarios. Fig 2 shows before Treat All, 40% (n = 142) of the analytical sample had initiated treatment 0–20 days before attending their first clinic date at a treatment site compared to 54% (n = 596) after Treat All. Table 2 further describes the final analytical sample which had similar characteristics to the initial sample with regards to the demographic variables and clinical outcomes assessed. The time to ART initiation also significantly decreased after Treat All (median 7 days vs. 0 days, p = 0.03).

**Table 2:** Demographic and clinical characteristics for the analytical sample of persons living with HIV (PLHIV) on ART.

Variables	Levels	Before Treat All		After Treat All	
		n	%	n	%
**Gender**					
	Female	169	47	578	53
	Male	187	53	521	47
**Age group at ART initiation/years**					
	Mean (SD)	40 (12.8)		39 (13.5)	
	< 15 years	4	1	6	1
	15–19	8	2	50	4
	20–39	170	48	555	51
	40+	173	49	487	44
**RHA^a^**					
	SERHA	194	55	568	52
	WRHA	100	28	323	29
	NERHA	33	9	111	10
	SRHA	29	8	97	9
**HIV stage at diagnosis ^b^**					
	median baseline CD4 result/cells/mm^3^ (IQR)	293.5 (338.3)		402 (392)	
	early diagnosis	141	40	590	54
	late diagnosis	215	60	427	39
	missing (excluded from analysis)	0	0	82	7
	median time to ART initiation/days ^c^ (IQR)	7 (109.2)		0 (56)	
**Viral load status after ART initiation^d^**					
	median first viral load result/copies/mL (IQR)	20 (827.3)		19 (157.5)	
	non-suppressed	87	24	222	20
	suppressed	269	76	877	80
	median time to first VL test/days^e^ (IQR)	154 (70)		159 (72.5)	

Characteristics of the analytical sample before Treat All in 2015–2016 (N = 356) and after Treat All in 2017–2019 (N = 1099).

a) RHA-Regional Health Authority, SERHA-Southeast Regional Health Authority, SRHA–Southern Regional Health Authority, NERHA–Northeast Regional Health Authority, WRHA–Western Regional Health Authority.

b) Based on baseline CD4 test results: Patients starting ART with CD4 cell count ≧350 cells/mm3 were characterized as achieving early diagnosis, while CD4 <350 cells/mm3 was defined as late diagnosis.

c) Time/days to ART initiation is the time from first clinic date to ART initiation.

d) Based on the first viral load test after initiating treatment, followed by categorization according to the first viral load test results at that time period (<1000 copies/mL versus ≧1000 copies/mL).

e) Time/days to VL test is the time from the ART initiation date to first VL test date.

Table 3 shows after Treat All, there was a significant decrease in late diagnosis across all categories and an increase in viral suppression across most categories except for 15–19 years and WRHA.

**Table 3:** Distribution of persons living with HIV (PLHIV) by outcome.

	Before Treat All						After Treat All					
Variables	late HIV diagnosis			suppressed viral load status			late HIV diagnosis			suppressed viral load status		
	n	%	p	n	%	p	n	%	p	n	%	p
**Gender**												
Female	89	52.7	0.006	122	72.2	0.199	190	35.6	<0.001	456	78.9	0.475
Male	126	67.4		147	78.6		237	49.1		421	80.8	
**Age group**												
<15	1	25.0	0.004	2	50.0	0.47	2	50	0.01	3	50	0.249
15–19	4	50.0		7	87.5		20	46.5		42	84.0	
20–39	88	51.8		124	72.9		188	36.6		438	78.9	
40+	121	69.9		136	78.6		216	47.4		393	80.7	
**RHA**												
SERHA	122	62.9	0.034	147	75.8	0.013	246	46.2	0.037	445	78.3	0.326
WRHA	51	51.0		84	84.0		118	37.9		267	82.7	
NERHA	19	57.6		20	60.6		34	35.4		91	82.0	
SRHA	23	79.3		18	62.1		29	37.2		74	76.3	

Distribution of the analytical sample by late HIV stage of diagnosis and suppressed viral load status, before Treat All in 2015–2016 and after Treat All in 2017–2019.

a) Percentages are of category row totals and have been rounded up.

b) RHA-Regional Health Authority, SERHA-Southeast Regional Health Authority, SRHA–Southern Regional Health Authority, NERHA–Northeast Regional Health Authority, WRHA–Western Regional Health Authority.

c) HIV stage at diagnosis based on initial CD4 test results: Patients starting ARV with CD4 cell count ≧350 cells/mm3 were characterized as achieving early diagnosis, while CD4 <350 cells/mm3 was defined as late diagnosis.

d) Viral load status based on the first viral load test date after initiating treatment, followed by categorization according to the first viral load test results at that time period (<1000 copies/mL versus ≥1000 copies/mL).

**Fig 3:**
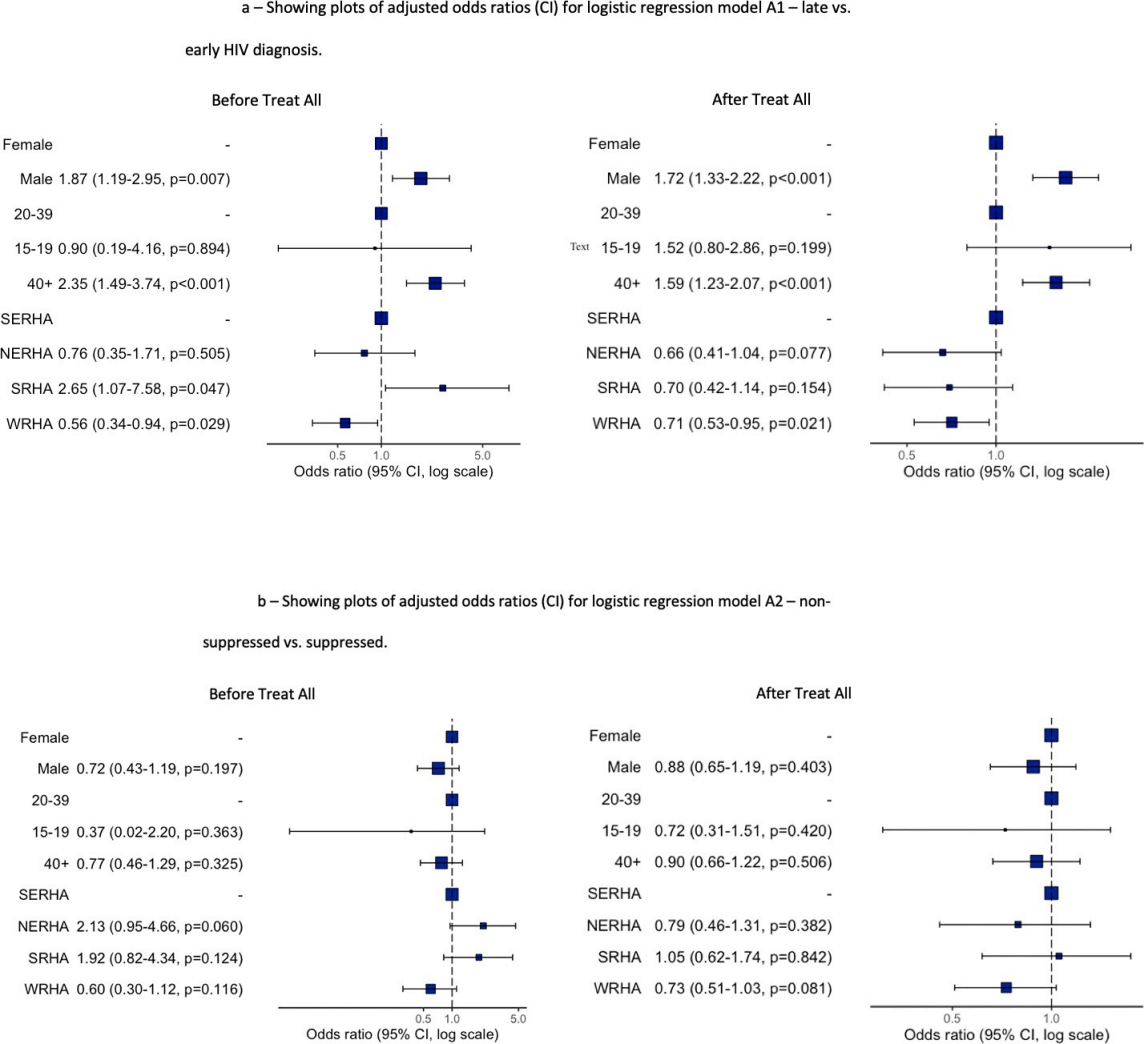
A. Plots of adjusted odds ratios (CI) for logistics regression model A1 before and after Treat All representing late vs. early HIV diagnosis. B. Plots of adjusted odds ratios (CI) for logistics regression model A2 before and after Treat All representing non suppressed viral load status vs. suppressed viral load status.

Fig 3A shows males were almost two times more likely to be diagnosed late compared to females before (aOR = 1.87, CI = 1.19–2.95) and after (aOR = 1.72, CI = 1.33–2.22) Treat All (Fig 3A). This was highly significant. Compared to 20–39 age group, persons 15–19 years (aOR = 0.90, CI = 0.19–4.16) were less likely to be diagnosed late before Treat All but more likely after Treat All (aOR = 1.52, CI = 0.80–2.86), while 40+ years were two times more likely to be diagnosed late before (aOR = 2.35, CI = 1.49–3.74) and after (aOR = 1.59, CI = 1.23–2.07) Treat All. Persons in SRHA (aOR = 2.65, CI = 1.07–7.58) had significantly higher odds of late diagnosis before Treat All, but lower odds after Treat All compared to SERHA. All other regions were associated with early diagnosis before and after Treat All. Fig 3B shows none of the demographic variables investigated were significantly associated with viral non-suppression.

Table 4 shows there was minimal decrease in persons diagnosed late and non-suppressed (28.6% to 26.7%) after Treat All, while the proportion diagnosed early and virally non-suppressed also changed minimally (16.7% to 16%).

**Table 4:** Number (n) and proportion (%) of virally suppressed and non-suppressed HIV treatment patients, by HIV stage at diagnosis, between 2015–2019.

	Before Treat All				After Treat All			
Variables	suppressed		non-suppressed		suppressed		non-suppressed	
	n	%	n	%	n	%	n	%
**HIV stage at diagnosis**								
**Late**	152	71.4	61	28.6	311	73.3	113	26.7
**Early**	115	83.3	23	16.7	494	84.0	94	16

HIV stage of diagnosis in the analytical sample by viral load status, before Treat All in 2015–2016 and after Treat All in 2017–2019.

a. Number in model before Treat All = 351, Number in model after Treat All = 1012.

b. HIV stage at diagnosis based on initial CD4 test results: Patients starting ARV with CD4 cell count ≧350 cells/mm3 were characterized as achieving early diagnosis, while CD4 <350 cells/mm3 was defined as late diagnosis.

c. Viral load status based on the first viral load test date after initiating treatment, followed by categorization according to the first viral load test results at that time period (<1000 copies/mL versus ≥1000 copies/mL).

Using Equation Model B, late diagnosis was associated with two times higher odds of viral non-suppression before (aOR = 2.01, CI = 1.19–3.49) and after Treat All (aOR = 1.91, CI = 1.40–2.60). This was highly significant.

## Discussion

One of the primary objectives of Treat All is to increase early treatment initiation [6]. Our results suggest this objective has been realized, as the time to ART initiation significantly decreased after Treat All in both the baseline and analytical samples. There was also an increase in PLHIV initiating ART 0–20 days before attending their first clinic date at a treatment site after Treat All, in the analytical sample. Jamaica’s national data indicates 23% of new HIV diagnoses occurs in hospitals through provider-initiated treatment and counselling (PITC) [1]. Our study results suggest persons are initiating treatment before linking to the primary care treatment sites, possibly while in hospital (or private care). The proportion of persons diagnosed at an early HIV stage also increased from 40% to 54%. This was further confirmed by an increase in the median baseline CD4 count from 302 cells/mm^3^ to 419 cells/mm^3.^ after Treat All.

Despite the overall decrease in late diagnosis, males were significantly more likely to be diagnosed late before and after the strategy was implemented compared to females. This is consistent with other studies reporting late diagnosis was more common in men than in women, attributed to poor health seeking behaviour [9, 21, 22]. HIV screening is also actively included with antenatal services in Jamaica [23], which facilitates more females being diagnosed. Persons 40+ years were also significantly more likely to be diagnosed late compared to 15–19 years and 20–39 years old. The study justifies the need for HIV screening and testing services to target males and persons over 40 years. However, targeting these specific populations for diagnosis should also include an assessment of their unique challenges.

The baseline sample showed a decrease in baseline viral load testing after Treat All (61% vs. 46%) and there was an increase in the uptake of viral load testing after 91–240 days on treatment. This may be explained by the implementation of new national testing guidelines in 2017, which stipulate a first viral load test should be administered 3–6 months after starting treatment [16]. This is also consistent with the Treat All strategy objectives to use routine viral load monitoring as the preferred approach to diagnose and confirm treatment failure, adherence and clinical management for PLHIV [10]. Currently, HIV viral load testing is only conducted at the National Laboratory. The National HIV Strategic Plan has outlined non-adherence to testing protocols and other laboratory-based challenges as impediments to viral suppression [24]. Other countries have shown that decentralizing viral load testing can increase uptake at the site level by increasing access points and improving convenience [25]. Jamaica could possibly explore decentralization to HIV treatment sites and partnership with private laboratories network but further analysis is required to more concretely determine how and to what extent laboratory challenges have affected testing uptake and other clinical outcomes.

The frequency of persons achieving viral suppression after initiating treatment increased only minimally after Treat All (76% to 80%). So, despite majority of persons diagnosed late before Treat All, most were still able to achieve suppression at the first viral load test. This may speak to the effect of early treatment initiation after diagnosis, as the median time to ART initiation was 7 days before Treat All.

Our study shows males and age groups 15–19 and 40+ years were more likely to be virally non-suppressed before and after Treat All. This again may be a result of early treatment. There was a change in the viral load status of PLHIV in NERHA, moving from two times higher odds of viral non-suppression before Treat All to lower odds after Treat All. Using Equation Model B, we also found late diagnosis was significantly associated with viral non-suppression at the first viral load test, both before and after Treat All. While, Treat All is intended to ensure immediate start on ART to improve health outcomes, viral suppression also depends on adherence to treatment. Persons who seek health care services late are more likely to demonstrate poor adherence in keeping with poor health behaviour. This study could not assess adherence, but national data shows Jamaica has only achieved 53% of the global adherence target [2], so even though treatment may be initiated at diagnosis, if it is not consistently adhered to the benefits are reduced.

Despite the difference in sample size between both scenarios, gender was mostly equally distributed and all four regional health authorities were sampled. While time periods sampled for the two scenarios varied (1 year before Treat All and 2 years after Treat All), the time intervals used for the relevant variables assessed were consistent. However, we acknowledge certain limitations of the data. The dataset was extracted from TSIS2 in February of 2020 and therefore, only provides a snapshot of the patients’ data. The dataset lacked HIV diagnosis dates, so our assessment of time to ART initiation used the time from first clinic date to ART initiation. This assumes the first clinic date is the first encounter with a treatment site and negates possible use for non-HIV related services. Viral load test results after the first test and treatment adherence is also not assessed. Therefore, it is assumed persons on treatment for 91–240 days are consistent in taking medication. Also, we were unable to assess deaths because persons found to be deceased are removed from the database. So, all persons in the sample are considered to be alive. To ensure there would be no overlap of the time periods, persons who were not yet eligible for a first viral load test, those who were eligible but did not receive a test (no test results) and those who received a first viral load test either before or after the study periods were all excluded from the sample. This reduced sample sizes in both scenarios. We recognize the possible effect of a smaller sample size, so the age group 15–19 years has been excluded in the final analysis because of the wider confidence intervals noted and we have used the categories with the largest samples as references in the regression analyses [18, 26]

## Conclusion

Overall, the time to ART initiation for PLHIV in Jamaica was significantly reduced after implementation of the Treat All Strategy. There was also a decrease in late diagnosis while viral load testing after treatment initiation and viral suppression for those on treatment increased. However, there is a need to examine the effect of laboratory based challenges on viral load testing uptake and the achievement of viral suppression as well as identify service delivery models to improve diagnosis for men and persons over 40 years.

## Supporting information

S1 File R code. (DOCX)

S2 File Data summary table. (DOCX)

## Abbreviations

ART: antiretroviral therapy
PLHIV: persons living with HIV
NERHA: Northeast Regional Health Authority
RHA: Regional Health Authority
SERHA: Southeast Regional Health Authority
SRHA: Southern Regional Health Authority
WRHA: Western Regional Health Authority
