# Correction: HIV program outcomes for Jamaica before and after “Treat All”: A population-based study using the national treatment services database

**DOI:** 10.1371/journal.pone.0303723

**Published:** 2024-05-09

**Authors:** Anya Cushnie, Ralf Reintjes, Susanna Lehtinen-Jacks, J. Peter Figueroa

There are errors in the Author Contributions. The correct contributions are:

**Conceptualization:** Anya Cushnie, Ralf Reintjes, Susanna Lehtinen-Jacks, J. Peter Figueroa.

**Data curation:** Anya Cushnie.

**Formal analysis:** Anya Cushnie.

**Investigation:** Anya Cushnie.

**Methodology:** Anya Cushnie, Ralf Reintjes, Susanna Lehtinen-Jacks, J. Peter Figueroa.

**Supervision:** Ralf Reintjes, Susanna Lehtinen-Jacks, J. Peter Figueroa.

**Validation:** Anya Cushnie.

**Visualization:** Anya Cushnie

**Writing–original draft:** Anya Cushnie

**Writing–review & editing:** Ralf Reintjes, Susanna Lehtinen-Jacks, J. Peter Figueroa.
