# Supplementary material for: HIV program outcomes for Jamaica before and after “Treat All”: A population-based study using the national treatment services database
Source: PLoS One. 2021 Aug 12;16(8):e0255781. doi: 10.1371/journal.pone.0255781 (PMC8360520; doi:10.1371/journal.pone.0255781)
Supplement: S1 File — (DOCX) [file pone.0255781.s001.docx]

attach(before)#load dataframes

attach(after)

library(tidyverse)

library(Hmisc)

library(summarytools)

#assess characteristics of full data set

view(dfSummary(before))

#assess time to first viral load test

hist(before$ `time to 1st VL`, col="gray", labels = TRUE, ylim=c(0, 100), breaks =30,xlab =

"time to first viral load test/days", ylab = "frequency",main="Time to first viral load test before Treat All (N=651)")

hist(after$ `time to 1st VL`, col="gray", labels = TRUE, ylim=c(0, 500), breaks =30,xlab =

"time to first viral load test/days", ylab = "frequency",main="Time to first viral load test after Treat All (N=2053)")

#subset data for 91-240 days on treatment (after 3 months)-final sample

beforesub <- subset(before, `time to 1st VL` >= 91 & `time to 1st VL`<= 240) #90 excluded. check minimum value. 356 total

aftersub <- subset(after, `time to 1st VL` >= 91 & `time to 1st VL`<=240)

#time to ART start

hist(beforesub$timetoARV, col="gray", labels = TRUE, xlim=c(-100, 500),ylim=c(0, 200), breaks=250,xlab =

"time to ARV initiation/days", ylab = "frequency",main="Histogram showing time to ARV initation before Treat All (N=356)")

hist(aftersub$timetoARV, col="gray", labels = TRUE, xlim=c(-100, 500),ylim=c(0,650), breaks=300,xlab =

"time to ARV initiation/days", ylab = "frequency",main="Histogram showing time to ARV initation after Treat All (N=1099)")

#bivariate analyis-before treat all

install.packages("scales")

library(finalfit)

explanatory = c("gender", "Agegrp", "RHA")

dependent = "CD4status"

beforesub %>% summary_factorlist(dependent, explanatory, p = TRUE, na_include = TRUE,

column = FALSE) -> table1

dependent2="Vlstatus"

beforesub %>% summary_factorlist(dependent2, explanatory, p = TRUE, na_include = TRUE,

column = FALSE) -> table2

#after treat all analysis

aftersub %>% summary_factorlist(dependent, explanatory, p = TRUE, na_include = TRUE,

column = FALSE) -> table3

aftersub %>% summary_factorlist(dependent2, explanatory, p = TRUE, na_include = TRUE,

column = FALSE) -> table4

library(rstan)

library(boot)

#remove ages 1-14 from age categories because of small sample sizes

beforesub2 <- subset(beforesub, ageatARV >14) #5 persons lost

aftersub2 <- subset(aftersub, ageatARV > 14) #7 persons lost

#CONVERT VARAIBLES TO FACTORS FOR REGRESSION ANALYSIS

fbefore <- mutate_at(beforesub2, vars(Agegrp, RHA, gender, CD4status,Vlstatus), as.factor)

fafter<-mutate_at(aftersub2, vars(Agegrp, RHA, gender, CD4status, Vlstatus), as.factor)

#check levels of factor

levels(fbefore$Agegrp)

#check reference categories for each group and re-level to most frequent value

fRHA<-relevel(fbefore$RHA, "SERHA")

FAGE<-relevel(fbefore$Agegrp,"20-39")

FGENDER<-relevel(fbefore$gender,"Female")

FCD4<-relevel(fbefore$CD4status,"EARLY")

#add new variables to data frame

fbefore$fRHA<-fRHA #column 30

fbefore$FAGE<-FAGE

fbefore$FGENDER<-FGENDER

fbefore$FCD4<-FCD4

#RELEVEL after Treat All dataset

AGE2<-relevel(fafter$Agegrp,"20-39")

GENDER2<-relevel(fafter$gender,"Female")

CD42<-relevel(fafter$CD4status,"EARLY")

RHA2<-relevel(fafter$RHA, "SERHA")

#add new columns to dataset

fafter$RHA2<-RHA2 #column 30

fafter$AGE2<-AGE2

fafter$GENDER2<-GENDER2

fafter$CD42<-CD42

#regression analysis for before treat all-Model A1-late vs early

explanatory2 = c("FGENDER", "FAGE", "fRHA")

dependent3 = "FCD4"

fbefore%>% finalfit(dependent3, explanatory2, metrics=TRUE) ->beforeCD4MODEL

print(beforeCD4MODEL)

#odds ratio plots

fbefore %>%

or_plot(dependent3, explanatory2,

breaks = c(0.5, 1, 5))

#Model A1-after Treat All-late vs early

explanatory3 = c("GENDER2", "AGE2", "RHA2")

dependent4 = "CD42"

fafter%>% finalfit(dependent4, explanatory3, metrics=TRUE) ->afterCD4MODEL

print(afterCD4MODEL)

fafter %>%

or_plot(dependent4, explanatory3,

breaks = c(0.5, 1, 5))

#Model A2 - non suppressed vs suppressed

#Before Treat All

explanatory2 = c("FGENDER", "FAGE", "fRHA")

dependent2 = "Vlstatus"

fbefore%>% finalfit(dependent2, explanatory2, metrics=TRUE) ->beforeVLMODEL

print(beforeVLMODEL)

fbefore %>%

or_plot(dependent2, explanatory2,

breaks = c(0.5, 1, 5))

#After Treat All

explanatory3 = c("GENDER2", "AGE2", "RHA2")

dependent2 = "Vlstatus"

fafter%>% finalfit(dependent2, explanatory3, metrics=TRUE) ->afterVLMODEL

print(afterVLMODEL)

fafter %>%

or_plot(dependent2, explanatory3,

breaks = c(0.5, 1, 5))

#Model B

explanatory4 = c("FCD4")

dependent2 = "Vlstatus"

fbefore%>% finalfit(dependent2, explanatory4, metrics=TRUE) ->beforeVLMODEL2

print(beforeVLMODEL2)

fbefore %>%

or_plot(dependent2, explanatory4,

breaks = c(0.5, 1, 5, 10, 20, 30))

explanatory5 = c("CD42")

dependent2 = "Vlstatus"

fafter%>% finalfit(dependent2, explanatory5, metrics=TRUE) ->afterVLMODEL2

print(afterVLMODEL2)

fafter %>%

or_plot(dependent2, explanatory5,

breaks = c(0.5, 1, 5))

#OR=1 Exposure does not affect odds of outcome

#OR>1 Exposure associated with higher odds of outcome

#OR<1 Exposure associated with lower odds of outcome

citation("finalfit")
