# Supplementary material for: HIV program outcomes for Jamaica before and after “Treat All”: A population-based study using the national treatment services database
Source: PLoS One. 2021 Aug 12;16(8):e0255781. doi: 10.1371/journal.pone.0255781 (PMC8360520; doi:10.1371/journal.pone.0255781)
Supplement: S2 File — (DOCX) [file pone.0255781.s002.docx]

**S2. Table of Data Summary**

| **Original variables** | **type** | **Before Treat All Policy**  **Sample population –** Persons starting HIV treatment between Jan 2015-December 2016 and received the first VL test after 91-240 days on ARV (see figure 1 for inclusion criteria).  **Analytical Sample = 356 observations** | **After “Treat All” Policy**  **Sample population –** Persons put on HIV  treatment between Jan 2017-December 2019  and had a first viral load test after 91-240 days on ARV.  **Analytical sample = 1099** |
| --- | --- | --- | --- |
| gender | categorical (male, female) | Females = 169 (47.5%), Males = 187 (52.5%) | \| Females – 578 \| ( \| 52.6% \| ) \| \| --- \| --- \| --- \| --- \| \| Males - 521 \| ( \| 47.4% \| ) \| |
| age at start of treatment/yrs | continuous (years) | Mean age (sd): 39.8 (12.8)yrs  min < med < max:  0.7 < 39.6 < 74.9   \| Age group \| n(%) \| \| --- \| --- \| \| 1-4years \| 2 (0.6%) \| \| 10-14years \| 2(0.6%) \| \| 15-19years \| 8(2.2%) \| \| 20-39years \| 170(47.9%) \| \| 40+years \| 173(48.7%) \| | Mean age (sd) : 38.8 (13.5)yrs  min < med < max:  0 < 37.5 < 91.7   \| Age group \| n(%) \| \| --- \| --- \| \| 0-4years \| 3(0.3%) \| \| 10-14years \| 3 (0.3%) \| \| 15-19years \| 50 (4.5%) \| \| 20-39years \| 555 (50.5%) \| \| 40+years \| 487 (44.4%) \| |
| RHA | categorical (Southeast, Southern, Northeast and Western) | SERHA- 194 (54.5%)  NERHA – 33 (9.3%)  SRHA-29 (8.2%)  WRHA-100 (28.1%) | SERHA- 568 (51.7%)  NERHA – 111 (10.1%)  SRHA-97 (8.8%)  WRHA-323 (29.4%) |
| treatment start date | date | min : 2015-01-06  med : 2015-10-06  max : 2016-09-08  range : 1y 8m 2d | min : 2017-01-01  med : 2018-05-22  max : 2019-09-16  range : 2y 8m 15d |
| first viral load test date | date | min : 2015-05-06  med : 2016-02-27  max : 2016-12-22  range : 1y 7m 16d | min : 2017-04-04  med : 2018-10-18  max : 2019-12-24  range : 2y 8m 20d |
| Time to first viral load test date/days | Time difference between ARV start date and viral load test date | Mean (sd) : 156.5 (42.6)days  min < med < max:  91 < 154 < 240  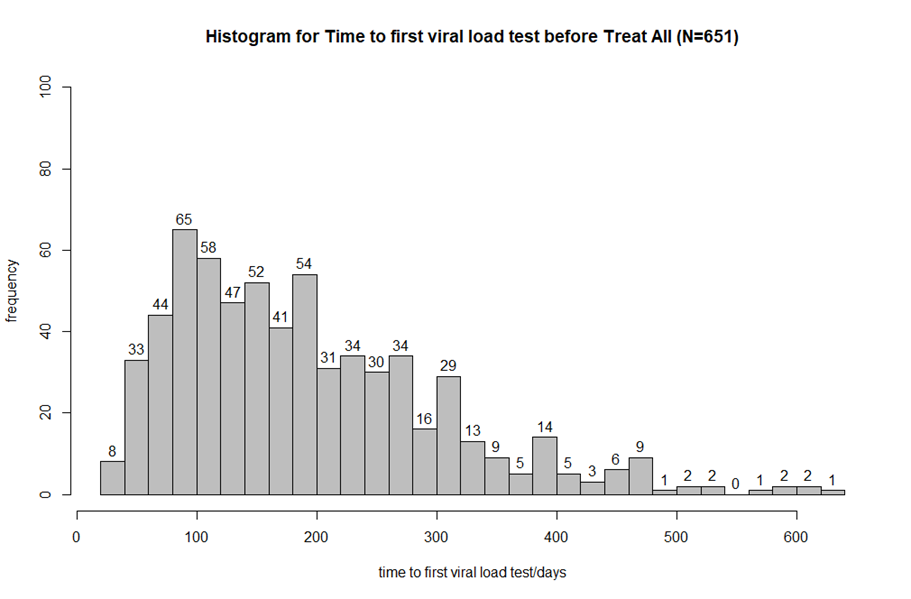 | Mean (sd) : 157.1 (42.6)days  min < med < max:  91 < 159 < 240  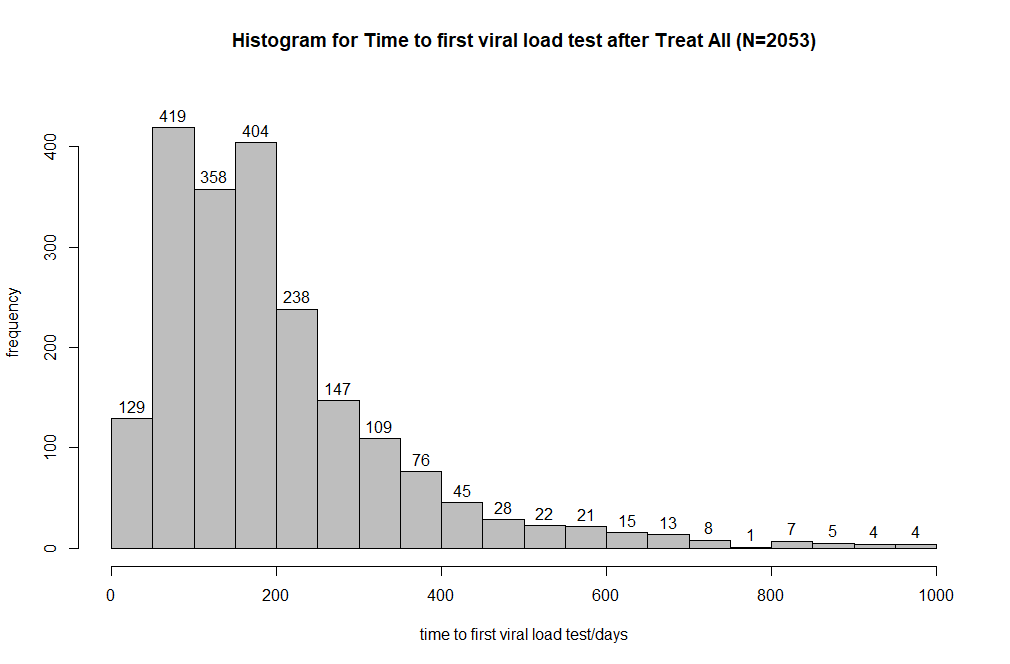 |
| first recorded viral load results | continuous (copies/mL) | Mean (sd) : 27359.8 (102572.7)  min < med < max:  19 < 20 < 898352 | Mean (sd) : 21882.7 (131071.5)  min < med < max:  19 < 19 < 2793819 |
| **derived variables** | **type** |  |  |
| first CD4 cell count  (HIV stage at diagnosis) | categorical (early: CD4 cell count $\geq350$ cells/mm^3^; or late: CD4 <350 cells/mm^3^) | \| Early = 141 \| ( \| 39.6% \| ) \| \| --- \| --- \| --- \| --- \| \| Late = 215 \| ( \| 60.4% \| ) \| \|  \|  \|  \|  \| | Early – 590 (58.0%)  Late - 427(42%) |
| Viral load status (suppressed: <1000 copies/mL at first VL test after starting ARV, vs. un-suppressed: $\geq$1000 copies/mL ) | categorical (suppressed, unsuppressed) | \| Suppressed = 269 \| ( \| 75.6% \| ) \| \| --- \| --- \| --- \| --- \| \| Unsuppressed = 87 \| ( \| 24.4% \| ) \| \|  \|  \|  \|  \| | \| Suppressed = 877 \| ( \| 79.8% \| ) \| \| --- \| --- \| --- \| --- \| \| Unsuppressed = 222 \| ( \| 20.2% \| ) \| \|  \|  \|  \|  \| |

**S2 Table: Univariable (OR) and multivariable (aOR) logistic regression assessing associations between demographic variables and stage at HIV diagnosis, among HIV treatment patients in Jamaica before and after Treat All, 2015-2019.**

|  | **Model A1 (late vs early diagnosis)** | | | | | |
| --- | --- | --- | --- | --- | --- | --- |
|  | Before Treat All | | | After Treat All | | |
|  | OR  (95%CI) | aOR  (95%CI) | p  value | OR  (95%CI) | aOR  (95%CI) | p  value |
| **Gender** |  |  |  |  |  |  |
| Male (vs female) | 1.85  (1.20-2.86) | 1.87  (1.19-2.95) | 0.007 | 1.73  (1.34-2.23) | 1.72  (1.33-2.22) | < 0.001 |
| **Age Group** |  |  |  |  |  |  |
| 20-39 years (ref) | 1 | 1 |  | 1 | 1 |  |
| 15-19 years | 0.93  (0.21-4.06) | 0.90  (0.19-4.16) | 0.894 | 1.50  (0.80-2.81) | 1.52  (0.80-2.86) | 0.199 |
| 40+ years | 2.17  (1.40-3.39) | 2.35  (1.49-3.74) | <0.001 | 1.56  (1.20-2.01) | 1.59  (1.23-2.07) | <0.001 |
| **RHA** |  |  |  |  |  |  |
| SERHA (ref) | 1 | 1 |  | 1 | 1 |  |
| WRHA | 0.64  (0.39-1.04) | 0.56  (0.34-0.94) | 0.029 | 0.70  (0.53-0.94) | 0.71  (0.53-0.95) | 0.021 |
| NERHA | 0.75  (0.35-1.63) | 0.76  (0.35-1.71) | 0.505 | 0.64  (0.40-1.00) | 0.66  (0.41-1.04) | 0.077 |
| SRHA | 2.25  (0.93-6.32) | 2.65  (1.07-7.58) | 0.047 | 0.69  (0.42-1.12) | 0.70  (0.42-1.14) | 0.154 |

Observations in model 1 before treat all = 351, after treat all = 1012

NERHA – Northeast Regional Health Authority, SERHA-Southeast Regional Health Authority, SRHA – Southern Regional Health Authority, WRHA – Western Regional Health Authority.

aOR= Adjusted odds ratios, CI= 95% confidence interval.

Reference categories were determined by largest proportions of sample

**S3 Table: Univariable (OR) and multivariable (aOR) logistic regression results assessing associations between demographic variables and viral load status, among HIV treatment patients in Jamaica, before and after “Treat All”.**

|  | **Model A2 (non-suppressed vs**  **suppressed)** | | | | | |
| --- | --- | --- | --- | --- | --- | --- |
|  | Before Treat All | | | After Treat All | | |
|  | OR  (95%CI) | aOR  (95%CI) | p  value | OR  (95%CI) | aOR (95%CI) | p  value |
| **Gender** |  |  |  |  |  |  |
| Male (vs female) | 0.67  (0.41-1.10) | 0.72  (0.43-1.19) | 0.197 | 0.89  (0.66-1.20) | 0.88  (0.65-1.19) | 0.403 |
| **Age Group** |  |  |  |  |  |  |
| 20-39 years (ref) | 1 | 1 |  | 1 | 1 |  |
| 15-19 years | 0.39  (0.02-2.25) | 0.37  (0.02-2.20) | 0.363 | 0.71  (0.30-1.48) | 0.72  (0.31-1.51) | 0.42 |
| 40+ years | 0.73  (0.44-1.20) | 0.77  (0.46-1.29) | 0.325 | 0.90  (0.66-1.21) | 0.90  (0.66-1.22) | 0.506 |
| **RHA** |  |  |  |  |  |  |
| SERHA (ref) | 1 | 1 |  | 1 |  |  |
| WRHA | 0.59  (0.30-1.10) | 0.60  (0.30-1.12) | 0.116 | 0.73  (0.51-1.04) | 0.73  (0.51-1.03) | 0.081 |
| NERHA | 2.24  (1.01-4.85) | 2.13  (0.95-4.66) | 0.060 | 0.80  (0.46-1.33) | 0.79  (0.46-1.31) | 0.382 |
| SRHA | 2.00  (0.86-4.49) | 1.92  (0.82-4.34) | 0.124 | 1.07  (0.63-1.77) | 1.05  (0.62-1.74) | 0.842 |

Observations in model 2 before Treat All =351, after Treat All =1092.

NERHA – Northeast Regional Health Authority, SERHA-Southeast Regional Health Authority, SRHA – Southern Regional Health Authority, WRHA – Western Regional Health Authority. aOR= Adjusted odds ratios, CI= 95% confidence interval. Reference categories were determined by largest proportions of sample.
